# Synthesis of 3-formyl-eudistomin U with anti-proliferation, anti-migration and apoptosis-promoting activities on melanoma cells

**DOI:** 10.1186/s13065-023-01102-1

**Published:** 2023-12-20

**Authors:** Jixiang Gao, Jinyi Liu, Tao Yu, Chenggong Xu, Hao Sun, Chunbo Lu, Wenjia Dan, Jiangkun Dai

**Affiliations:** 1 School of Life Science and Technology, Weifang Medical University, Weifang, Shandong Province 261053 China; 2 Central Hospital Affiliated to Shandong First Medical University, Jinan, Shandong Province 250100 China

**Keywords:** Eudistomin U, Synthesis, Anti-melanoma, Transcriptome, DNA

## Abstract

The discovery of new lead skeleton against melanoma are urgently needed due to its highly malignant and mortality. Herein, a new molecular entity (EU-5) derived from eudistomin U was synthesized with total yield of 46%, which displayed potent activity against malignant melanoma A375 cells (IC_50_ = 4.4 *µ*M), no hemolytic toxicity and good physicochemical properties *in silico*. Colony formation and cell cycle arrest assays revealed that EU-5 suppressed cell proliferation by causing cell cycle arrest at G0/G1 phase. Wound healing and transwell assays suggested that EU-5 could effectively inhibit migration of A375 cells in a dose-dependent manner. Calcein-AM/PI staining, Annexin V-FITC/PI apoptosis detection, mitochondrial membrane potential (MMP), reactive oxygen species (ROS), transcriptomics, quantitative real‑time polymerase chain reaction (qRT‑PCR), spectrometric titration and molecular docking assays indicated that EU-5 could activate p53 signaling pathway and trigger mitochondria-mediated cell apoptosis. Taken together, this study provided a promising lead structure for the design of a new generation of anti-melanoma drugs.

**Supplementary Information:**

The online version contains supplementary material available at 10.1186/s13065-023-01102-1.

## Introduction

Malignant melanoma with highly aggressive and metastatic is a high-mortality skin cancer [1]. Unfortunately, its incidence and drug resistance increased rapidly over the last decades. According to the latest statistics in 2020, there were more than 324,000 new cases of melanoma worldwide [2]. At present, surgery, chemotherapy, radiotherapy, targeted therapy and immunotherapy are the main treatment strategy for malignant melanoma in clinical practice [3]. In particular, traditional chemotherapy drugs are still the mainstay of treatment in most areas of the world due to its low cost [4]. In addition, immunotherapy and targeted drugs are also need to be supplemented with chemotherapy drugs due to emerging resistances and strong side effects [5]. Therefore, the discovery of new inhibitors against malignant melanoma are urgently needed.

Natural products have been an important source of antitumor drugs. Statistics showed that 84.3% (n = 156) of FDA approved new small-molecule antitumor drugs from 1981 to 2019 are derived from natural products, such as paclitaxel and vinblastine [6]. *β*-Carboline alkaloids have exhibited good antitumor activities by various mechanisms of action such as inhibition of sirtuin 5, kinesin-5 protein and proliferation, blocking of mitotic cell division, DNA intercalation and so on [7]. Eudistomin U, a member of *β*-carbolines, initially isolated from Caribbean ascidian *Lissoclinum fragile* in 1994, has showed antitumor activities against C19 leukemia cells (IC_50_ = 15.6 *µ*g/mL), CaOV3 ovarian cells (IC_50_ = 24.9 *µ*g/mL) and WM266-4 melanoma cells (IC_50_ = 27.5 *µ*g/mL, ~ 88.42 *µ*M) [8]. Moreover, previous study indicated than eudistomin U might act on DNA-related targets just like the natural antitumor drugs mitomycin and daunorubicin [9].

In our previous study, we focused on the construction of *β*-carboline compounds library including harmane, canthin-6-one and eudistomin U skeletons [10–12]. Herein, we tried to screen the antitumor activity of our library. Interestingly, a new compound 3-formyl-eudistomin U (EU-5) exhibited promising antitumor activity against A375 malignant melanoma cells (IC_50_ = 4.4 *µ*M), which was 20-fold more than eudistomin U against WM266-4 melanoma cells. In this work, we reported the synthesis, anti-melanoma activity and mechanism of action of EU-5, which will provide theoretical support for the design of a new generation of anti-melanoma drugs based on 3-formyl-eudistomin U skeleton.

## Results and discussion

### Chemistry

**Scheme 1:**
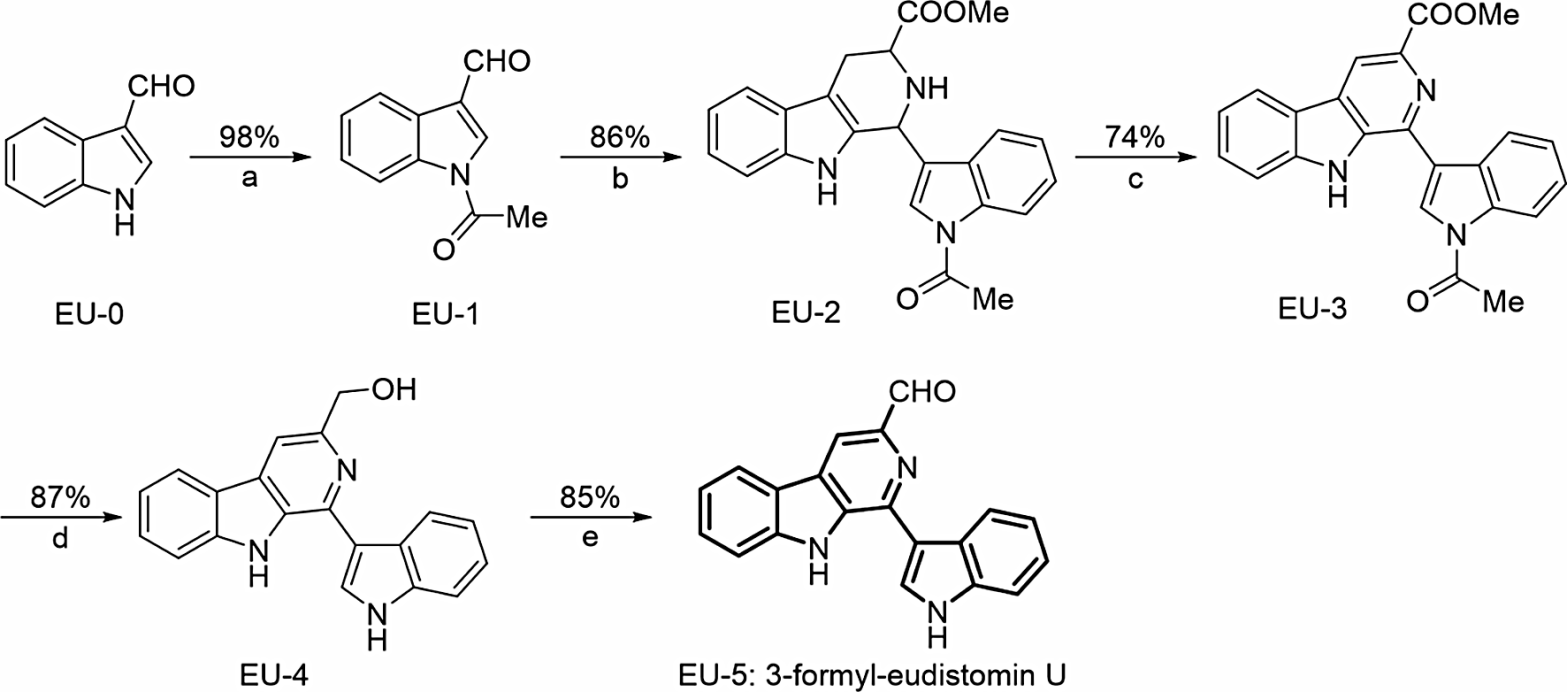
Synthesis of 3-formyl-eudistomin U. (**a**) Ac_2_O, Et_3_N, DMAP, DCM, 0 ^o^C ~ rt; (**b**) *i*, Trp-COOMe, methylbenzene, reflux; *ii*, DCM, TFA, 0 ^o^C ~ rt; (**c**) Pd/C, xylene, 150 ^o^C; (**d**) NaBH_4_, CaCl_2_, anhydrous EtOH, rt; (**e**) Dess-Martin periodinane, anhydrous DCM, 0 ^o^C ~ rt

In this work, we reported a synthetic pathway of 3-formyl-eudistomin U with 1*H*-indole-3-carbaldehyde as the starting material (Scheme 1). Previously, we have described the synthesis of EU-3 by continuously acylation, Pictet-Spengler condensation and oxidation reaction with total yield of 62.4% [11]. Subsequently, EU-4 was prepared by using NaBH_4_/CaCl_2_ system with anhydrous EtOH as the solvent (yield = 87%). Finally, Dess-Martin periodinane was used to prepare 3-formyl-eudistomin U (EU-5) with anhydrous DCM as the solvent (yield = 85%).

The structures of all synthesized compounds were confirmed by NMR and HRMS, which displayed in Figs. S1 and S2. The signals of aldehyde group of EU-5 were detected around *δ* 10.25 ppm (^1^H NMR) and *δ* 193.11 ppm (^13^C NMR). Moreover, the hydrogen atoms of aromatic ring were all founded from *δ* 7.20 ppm to *δ* 8.77 ppm in the ^1^H NMR spectra. In addition, the HRMS signal of EU-5 was detected at 312.1133 Da with adduct type of [M + H]^+^, which conformed to the theoretical value of 312.1131 Da (error < 5 ppm).

### In vitro antitumor activity, toxicity and physicochemical properties

**Fig. 1:**
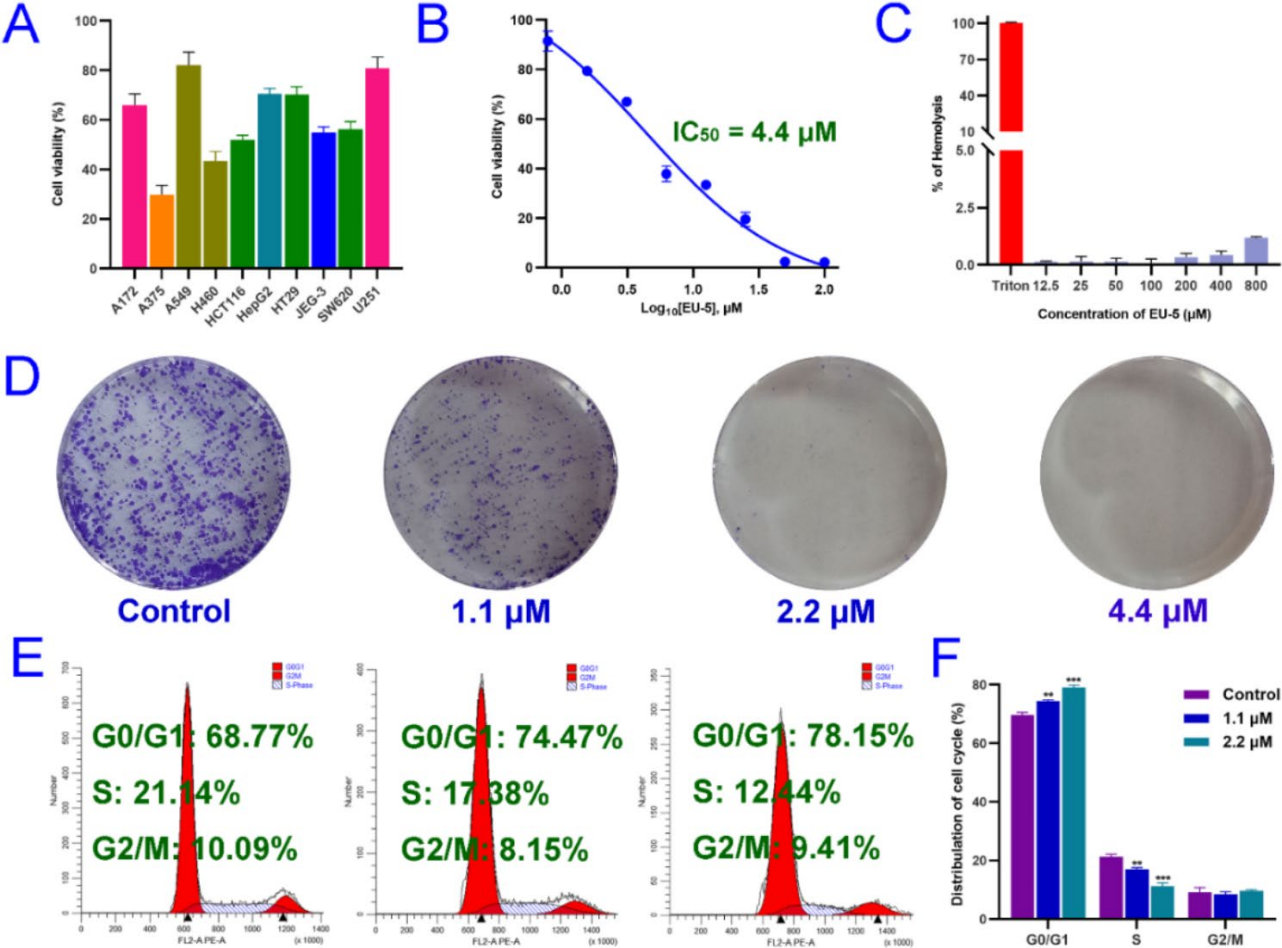
(**A**) The relative viability of ten kinds of tumor cells after treatment with 10 *µ*M of EU-5. (**B**) The in vitro antitumor activity of EU-5 against A375 cells. (**C**) Hemolytic toxicity of EU-5 against RBCs. (**D**) Colony formation of A375 cells after treatment with indicated concentrations of EU-5 for 7 days. (**E**) Cell cycle distribution of A375 cells after treatment with indicated concentrations of EU-5. (**F**) Cell cycle analysis by histograms, **P < 0.01, ***P < 0.001 vs. control group

As depicted in Fig. 1A, EU-5 exhibited broad-spectrum antitumor effect against the tested cell lines with cell viability percent from 29.9 to 82.1%. The growth of A375 cells was not affected by the negative control. In particular, EU-5 showed remarkable antitumor activity against A375 malignant melanoma cells (IC_50_ = 4.4 *µ*M) (Fig. 1B), which was comparable to the positive control cisplatin (IC_50_ = 4.98 *µ*M, Fig. S3). As depicted in Fig. S4, notably, EU-5 also exhibited similar cytotoxicity trends with cisplatin against human normal trophoblastic cells (HTR-8). Hemolysis is an important index in drug discovery, which could cause organ damage due to release of hemoglobin and hinder direct intravenous administration [13]. As displayed in Fig. 1C, no obvious hemolysis was observed, indicated the biosafety of the scaffold. To further assess the druggability of EU-5, its physicochemical properties were predicted by using online server SwissADME [14]. As shown in Table S1, the physicochemical descriptors of EU-5 conformed to the Lipinski’s rules, indicating good druggability. Moreover, it possessed high gastrointestinal absorption and no inhibition of cytochrome P450 2D6 (CYP2D6), ensuring the normal progress of drug metabolism. High expression of P-glycoprotein (P-gp) is closely related to tumor drug resistance [15]. Interestingly, EU-5 was calculated as an inhibitor for P-gp, which was beneficial for malignant melanoma therapy. Although compound EU-5 showed similar side effects on normal cells as cisplatin, the above results are still very inspiring because the issue can be solved through future structural modification or drug delivery system. The skeleton of EU-5 with outstanding activity and simple structure could be used as a lead structure to discover anti-melanoma drug.

### Antiproliferative analysis

Encouraged by the potential of EU-5, we further explored its mechanism of action. The ability of forming colonies is generally considered an essential feature of cancer cell malignancy [16]. As depicted in Fig. 1D, the number and size of colonies reduced significantly with increasing concentrations of EU-5. As shown in Fig. S5, compared to control group, EU-5 significantly inhibited A375 cell proliferation as well as colony formation ability (*p* < 0.05). Almost complete loss of clonogenic survival of the A375 cells was presented after treatment with 4.4 *µ*M of EU-5. As depicted in Fig. S6, the EdU assay was performed to further investigate the proliferation inhibition activity of EU-5 against A375 cells [17]. Compared to control group, the significant proliferation reduction in the treated group (4.4 *µ*M) was presented. The above results revealed that EU-5 could suppress the proliferation of A375 cells.

Generally, cell cycle progression is closely related to cell proliferation [16]. As shown in Fig. 1E, A375 cells in control group presented a normal cell cycle distribution with the ratio of 68.77% (G0/G1), 21.14% (S) and 10.09% (10.09%), respectively. After treated by EU-5, the portions of A375 cells in G0/G1 phase increased remarkably and in S phase decreased significantly with a dose-dependent manner (Fig. 1F). The above results suggested that EU-5 arrested A375 cell cycle at G0/G1 phase.

### Migration inhibition analysis

**Fig. 2:**
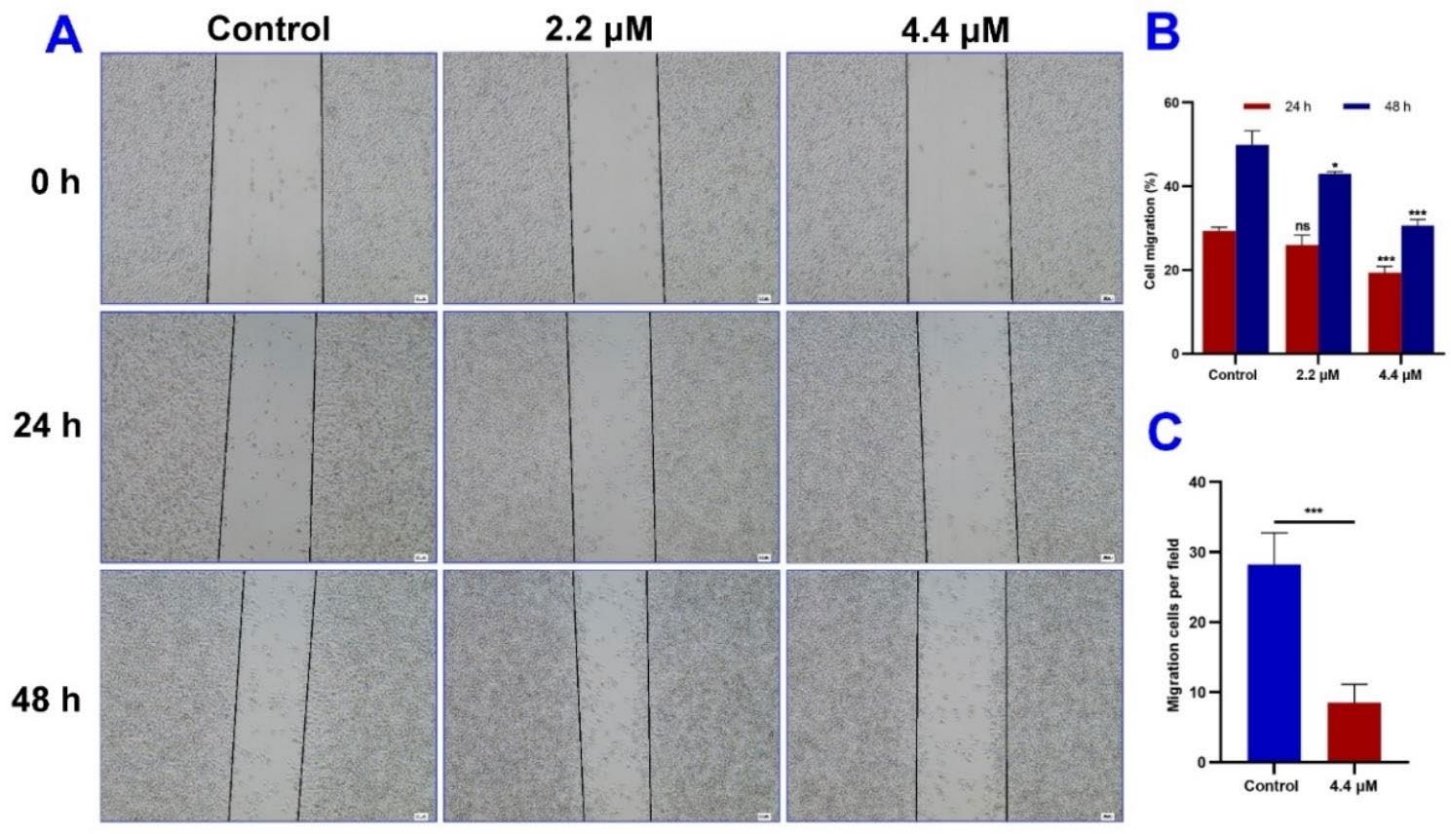
(**A**) The wound healing assay was performed using A375 cells treated by indicated concentrations of EU-5 for 24 and 48 h, scale bar = 100 *µ*m. (**B**) The wound closure area analysis. (**C**) The transwell assay was performed using A375 cells treated by 4.4 *µ*M of EU-5 for 24 h, ***P < 0.001 vs. control group

Migration is an important index of cancer metastasis and malignancy, especially for malignant melanoma [18]. As depicted in Fig. 2A, B and a wound healing assay was carried out to evaluate the migration inhibition ability of EU-5. Obvious wound closure of the control group was presented in a time-dependent manner (0, 24 and 48 h). By contrast, EU-5 significantly suppressed wound closure of A375 cells in a dose-dependent manner (2.2 and 4.4 *µ*M) at 48 h. After treated by 4.4 *µ*M of EU-5, the ratio of cell migration reduced 9.93% and 19.32% at 24 and 48 h, respectively. Additionally, transwell assay revealed that the number of cells penetrating the chamber reduced 69.91% after treated by 4.4 *µ*M of EU-5 for 24 h (Fig. 2C). Taken together, the results indicated that EU-5 could inhibit migration of A375 malignant melanoma cells.

### Cell apoptosis analysis

**Fig. 3:**
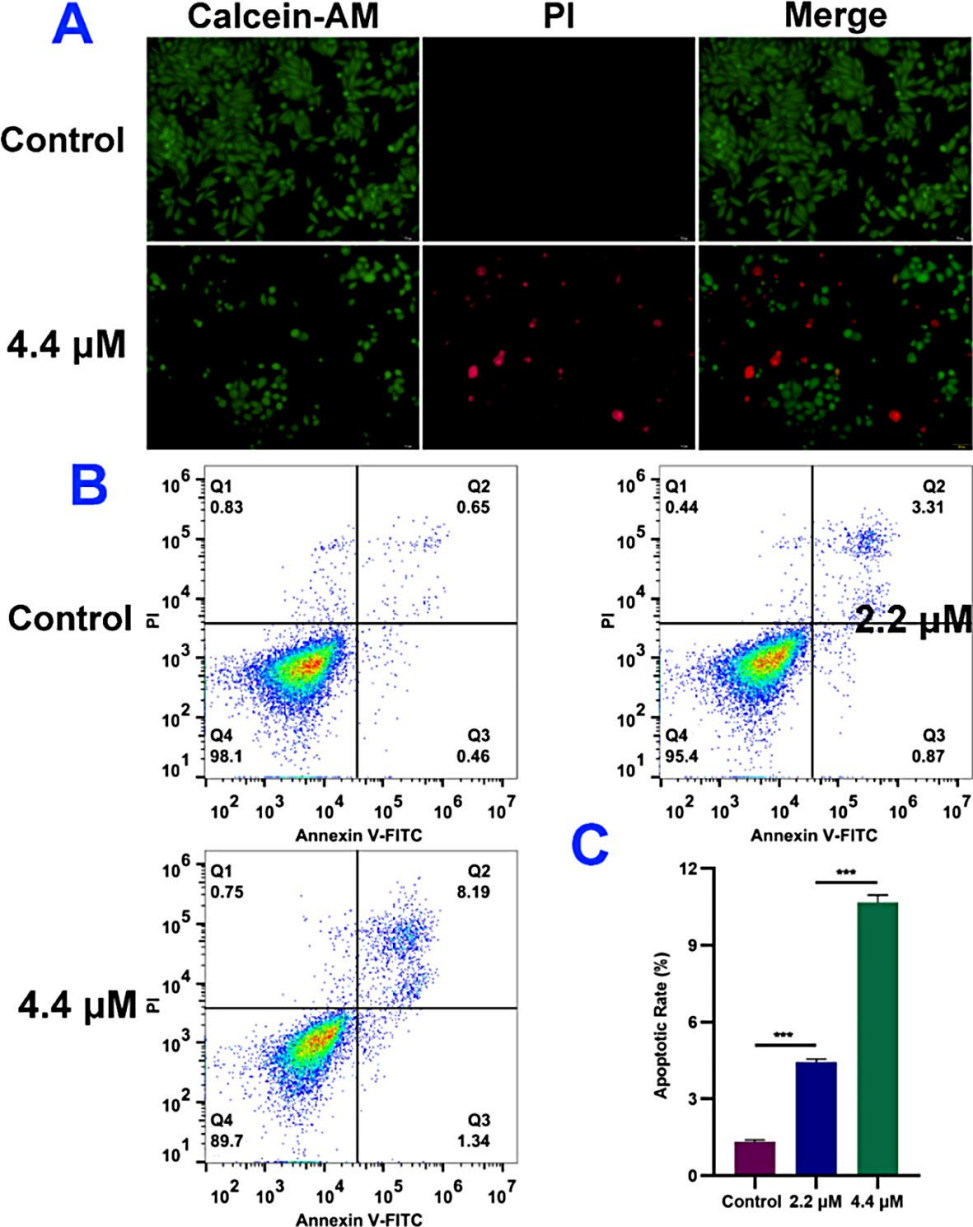
(**A**) Morphology of A375 cells treated by 4.4 *µ*M of EU-5 and stained with Calcein-AM/PI, scale bar = 50 *µ*m. (**B**) Annexin V-FITC/PI staining and flow cytometric analysis of apoptotic A375 cells treated by indicated concentrations of EU-5. (**C**) Apoptotic rate analysis by histograms, ***P < 0.001 vs. control group

Calcein acetoxymethyl ester/propidium iodide (Calcein-AM/PI) staining assay which can differentiate living and dead cells, was performed to observe A375 cell morphology [19]. As shown in Fig. 3A, living cells labeled with calcein (green fluorescence) have complete cell morphology and dense cells with normal physiology. After treated by 4.4 *µ*M of EU-5, cell proliferation rate was significantly reduced and the dead cells were labeled with PI (red fluorescence). As we all known, apoptosis and necrosis are two patterns of cell death [20]. Then A375 cells were double stained with Annexin V-FITC/PI to investigate the patterns of cell death [21]. As depicted in Fig. 3B and C, the total percentage of early and late apoptosis increased in a dose-dependent manner. Specifically, the apoptosis rate increased to 10.96% from 1.34% after treated by 4.4 *µ*M of EU-5. Notably, necrotic cells were little and there was no significant change after treatment.

**Fig. 4:**
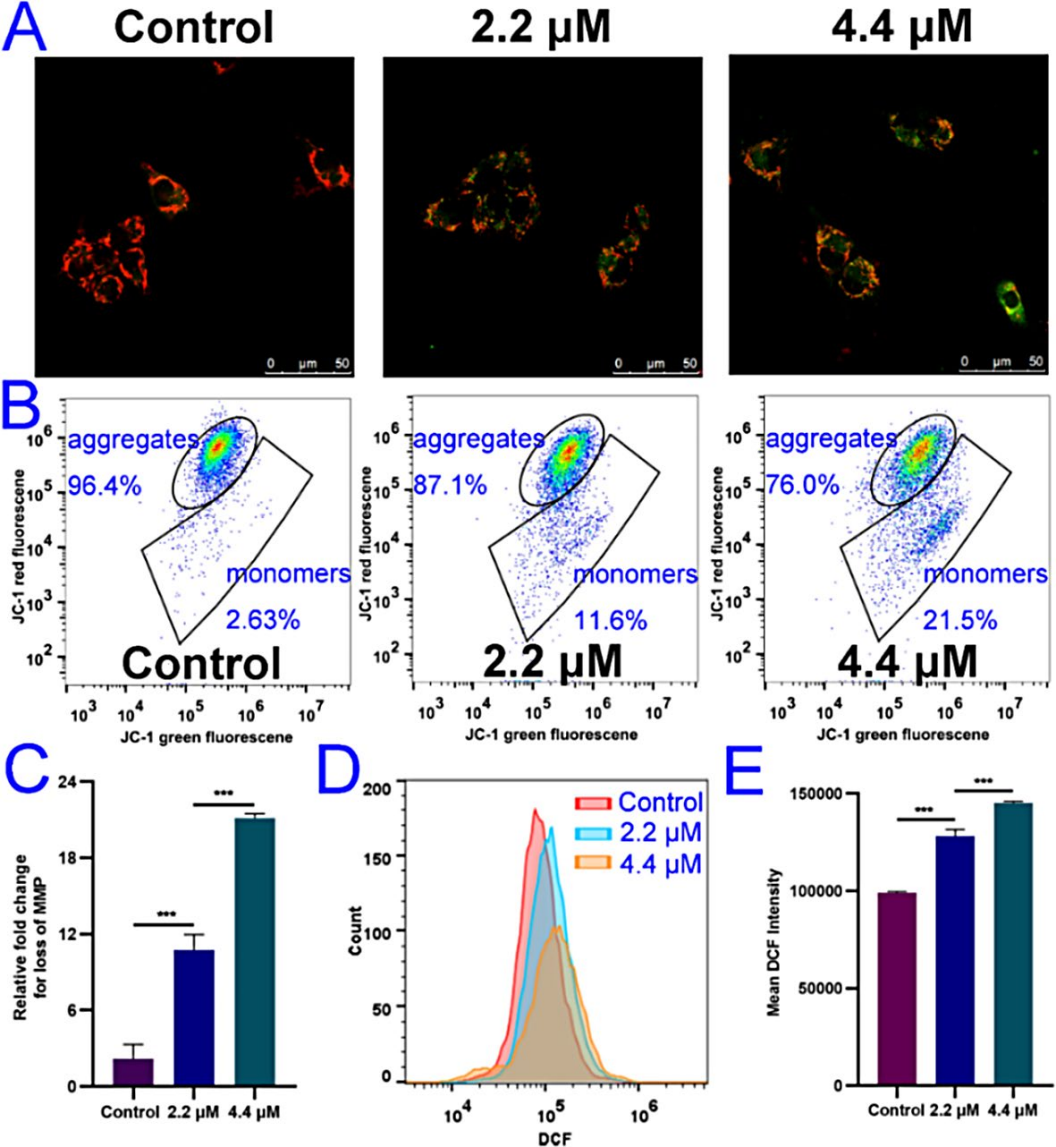
(**A**) The effect of indicated concentrations of EU-5 on MMP in A375 cells stained with JC-1, scale bar = 50 *µ*m. (**B**) MMP analysis by flow cytometry. (**C**) The loss of MMP by histograms. (**D**) The effect of indicated concentrations of EU-5 on intracellular ROS level in A375 cells stained with DCFH-DA by flow cytometric analysis. (**E**) The mean fluorescence intensity analysis by histograms, ***P < 0.001 vs. control group

Mitochondria-mediated caspase activation pathway triggered by DNA damage, overexpression of intracellular reactive oxygen species (ROS), etc., is a major apoptotic pathway, which is classically characterized by mitochondrial outer membrane permeabilization [22]. JC-1 (5,5′,6,6′-tetrachloro-1,1′,3,3′-tetraethyl-imidacarbocyanine) dye is widely used to detect mitochondrial membrane potential (MMP, ΔΨm), which present red fluorescence (aggregates) for normal MMP [23]. By contrast, green fluorescence (monomers) will increase when MMP reduction which can cause release of cytochrome *c* into the cytoplasm to activate caspases. As depicted in Fig. 4A, obvious red fluorescence loss while green fluorescence accumulation with increasing concentration of EU-5 was observed. As depicted in Fig. 4B, the proportion of JC-1 aggregates decreased from 96.4 to 87.1% and 76.0%, respectively. And the proportion of JC-1 monomer increased from 2.63 to 11.6% and 21.5%, respectively. MMP of A375 cells remarkably decreased after treatment with EU-5 (Fig. 4C). The results suggested that EU-5 could trigger the apoptosis by mitochondria pathway.

The overexpression of intracellular ROS can induce oxidative stress damage such as lipids and DNA, and further result to cell apoptosis. Previous study indicated that *β*-carboline alkaloids could increase the intracellular ROS level in A549 cells and thus triggered the cell apoptosis [22]. Mitochondria is the main site of reactive oxygen species production. The increase of ROS can lead to the opening of the mitochondrial membrane permeability transporter pore, which causes the decrease of mitochondrial transmembrane potential, the release of cytochrome *c*, and the activation of series of caspases enzymes, inducing the occurrence of cell apoptosis [24]. So, we detected the level of intracellular ROS in this work. 2′,7′-Dichlorofluorescein diacetate (DCFH-DA) can be oxidized by intracellular ROS to generate 2′,7′-dichlorofluorescein (DCF), which emit green fluorescence [21]. As depicted in Fig. 4D and E, the mean fluorescence intensity of DCF significantly increased after treatment by EU-5. Specifically, 1.47-fold increase of the DCF intensity was presented after treated by 4.4 *µ*M of EU-5 compared with the control group. The results suggested that EU-5 could remarkably increase the level of intracellular ROS, which might cause the alteration of the mitochondrial membrane potential and thus trigger the mitochondria-mediated cell apoptosis.

### Transcriptome analysis

**Fig. 5:**
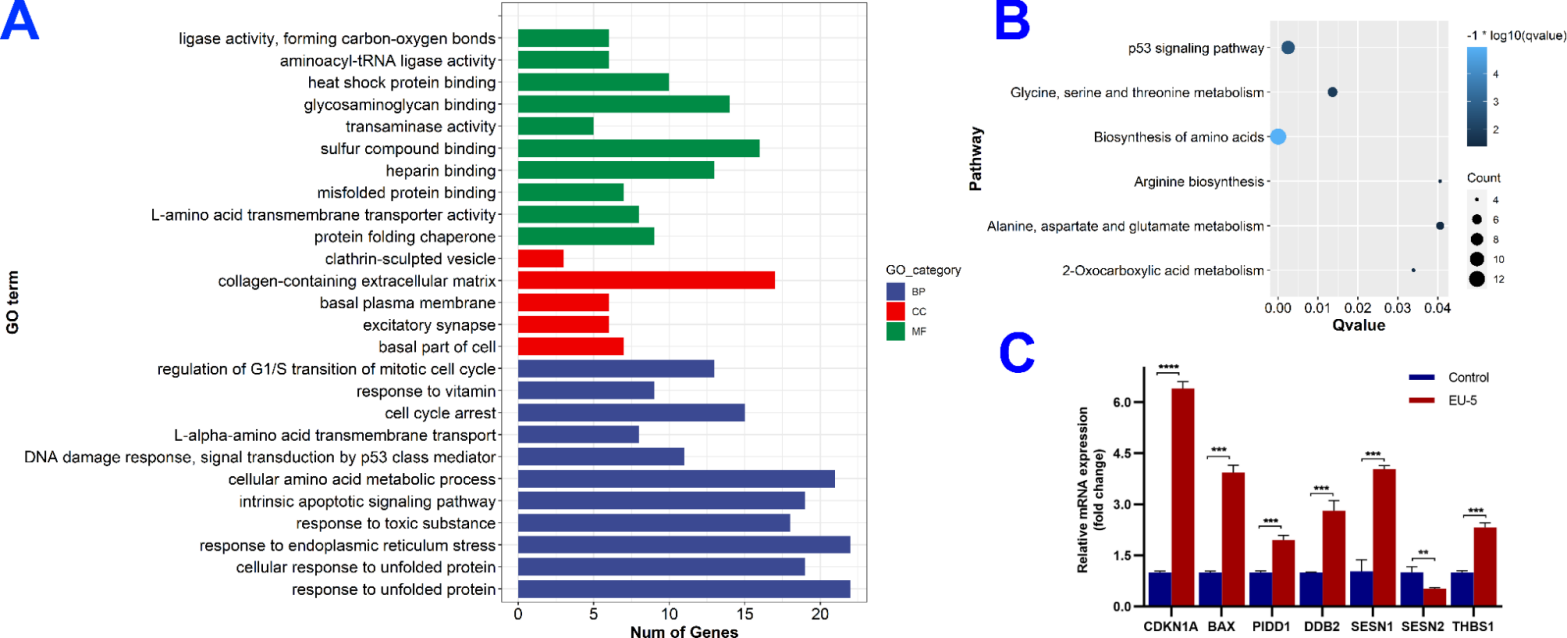
(**A**) The top enriched GO terms. (**B**) The enriched KEGG pathways. (**C**) RT-qPCR validation of representative DEGs from p53 signaling pathway, **P < 0.01, ***P < 0.001, ****P < 0.0001 vs. control group

Transcriptome sequencing analysis was further performed to investigate the mechanism from a global perspective [25]. Total of 23,117 genes were changed for A375 cells after treated by 4.4 *µ*M of EU-5. As shown in Fig. S7, 373 differentially expressed genes (DEGs) including 175 up-regulated genes and 198 down-regulated genes, were confirmed with cut-off criteria of |log_2_FC| ≥1 and Padj < 0.05. Then the gene ontology (GO) functional annotation and Kyoto Encyclopedia of Genes and Genomes (KEGG) enrichment analysis were performed by using clusterProfiler. As shown in Table S2 and Fig. 5A and 102 GO terms including 80 subclasses of biological processes, 5 subclasses of cellular component, and 17 subclasses of molecular function were annotated. Some types of biological processes related to antiproliferative activity were enriched which included cell cycle arrest, regulation of G1/S transition of mitotic cell cycle, mitotic.

DNA integrity checkpoint, G1/S transition of mitotic cell cycle, cell cycle G1/S phase transition, etc. Moreover, many GO terms related to cell apoptosis were presented, such as response to unfolded protein, response to endoplasmic reticulum stress, positive regulation of release of cytochrome *c* from mitochondria and intrinsic apoptotic signaling pathway, which conformed to the result of cell apoptosis analysis.

As shown in Table S3 and Fig. 5B, p53 signaling pathway, closely associated with cell cycle arrest and apoptosis [25], was significantly enriched (qvalue = 0.0025). Quantitative real‑time polymerase chain reaction (qRT‑PCR) was used to verify the results of RNA sequencing [26]. As shown in Fig. 5C, seven representative genes of p53 signaling pathway including *CDKN1A*, *BAX*, *PIDD1*, *DDB2*, *SESN1*, *THBS1*, and *SESN2*, were selected as target genes. Generally, the RT-qPCR data was consistent with the RNA sequencing result, indicating the credible and accurate of transcriptome. As we all known, the p53 protein is activated when cells suffer DNA damage, which further triggers a series of signal transduction events [27]. Simultaneously, the transcription level of *DDB2* gene will increase accordingly and participate in the recognition of damaged DNA [28]. The p53 protein will regulate the transcription of *CDKN1A* gene which can inhibit the activity of cyclin-dependent kinases (CDK), thereby causing the arrest of cell cycle in G1 phase [29]. When DNA damage cannot be repaired, the expression of *BAX*, *PMAIP1*, etc. will be activated, further triggering the mitochondria-mediated cell apoptosis pathway [30].

**Fig. 6:**
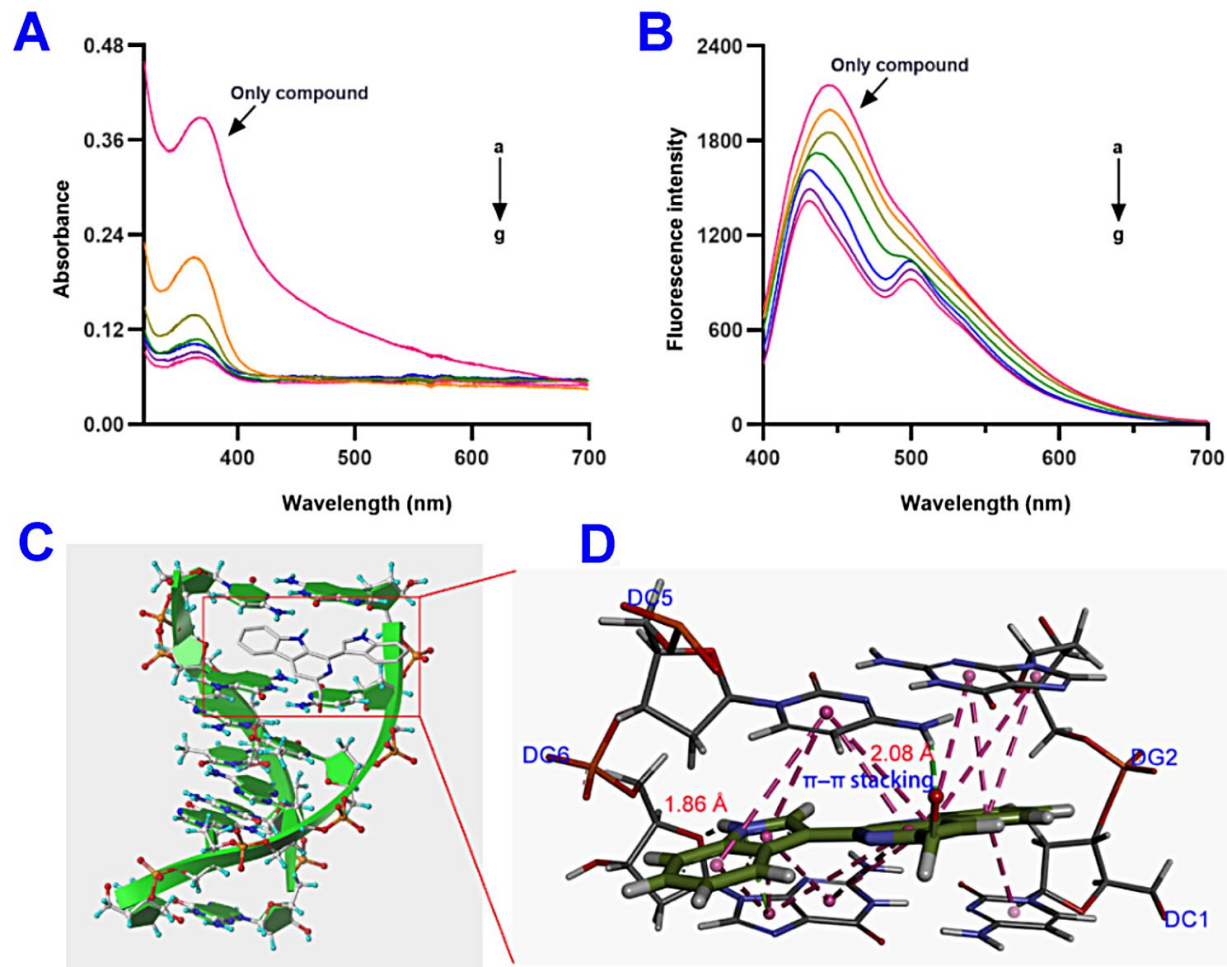
(**A**) UV spectra of EU-5 (5 × 10^− 5^ M) in the presence of indicated concentrations of DNA (0 − 3 × 10^− 5^ M). (**B**) Fluorescence spectra of EU-5 (5 × 10^− 5^ M) in the presence of indicated concentrations of DNA (0 − 3 × 10^− 5^ M). (**C**) Docking diagram of DNA and EU-5. (**D**) Interaction analysis in the active cavity

Previous studies have reported that carboline skeleton could directly attack the DNA related targets by groove bonding and intercalation between the base pairs to achieve antitumor effect [31]. In the present study, UV absorption and fluorescence spectroscopy were performed to investigate the binding mechanism with calf thymus DNA (ctDNA) as a model [32]. As shown in Fig. 6A, obvious blue shift and hypochromic effects are presented with the increasing concentration of ctDNA, indicating the change in conformations in the DNA helix. In addition, fluorescence quenching phenomenon and formation of new peaks at 500 nm suggested the generation of new DNA‒EU-5 complex by intercalation between the base pairs (Fig. 6B). Molecular docking study was further performed to visually investigate the binding mode [33]. As shown in Fig. 6C and D, EU-5 intercalated into DNA bases mainly by π‒π stacked interactions. Moreover, the aldehyde group and secondary amine fragment of EU-5 were adjacent to DC5 and DG6, respectively, forming two strong hydrogen bonds with 2.08 and 1.86 Å. The above results revealed that EU-5 could directly target DNA, causing DNA damage and disrupting its function.

## Experimental

### Materials and instruments

All chemical and biological reagents are commercially available. The tested cell lines including lung cancer cells (A549, H460), hepatoma cells (HepG2), glioma cells (A172, U251), melanoma cells (A375), colon cancer cells (HCT116, HT29, SW620), chorionic carcinoma cells (JEG-3) and human normal trophoblastic cells (HTR-8) were obtained from Cell Bank of Chinese Academy of Sciences. NMR spectra were recorded using a 500 MHz Bruker spectrometer. HR-MS spectra were detected by AB SCIEX Triple TOF 5600^+^ spectrometer. Flow cytometry analysis was performed by the BD Accuri™ C6 Plus instrument. Fluorescence images were obtained using the Leica TCS SP8, Nikon Eclipse Ti-s and Olympus IX73 instruments. Ultraviolet (UV) spectra were recorded by a UV-8000 spectrometer (METASH). Fluorescence spectra were recorded using a F-4600 spectrometer (Hitachi). Molecular docking study was performed by Sybyl-X 2.0 software.

### General procedure for synthesis of EU-5

*Chemical synthesis of EU-1.* To a stirred solution of 1*H*-indole-3-carbaldehyde (4.355 g, 30 mmol) in dry dichloromethane (100 mL) under ice bath was added 4-dimethylaminopyridine (122 mg, 1 mmol) and triethylamine (10.5 mL, 75 mmol). Subsequently, the acetic anhydride (4.23 mL, 45 mmol) was slowly added in batches. Then the reaction system was stirred at room temperature until the precipitate formed and complete conversion. The white slurry was filtered and the filtrate was recrystallized by cyclohexane and dichloromethane to afford compound EU-1 (white solid, 5.502 g, yield 98%). m.p. 165.7 − 167.5 ^o^C; ^1^H NMR (500 MHz, CDCl_3_) *δ* 10.13 (s), 8.41 (d, *J* = 10 Hz, 1H), 8.28 (d, *J* = 10 Hz, 1H), 8.08 (s, 1H), 7.49–7.42 (m, 2 H), 2.76 (s, 3 H); ^13^C NMR (125 MHz, CDCl_3_) *δ* 185.62, 168.57, 136.38, 135.19, 126.87, 126.06, 125.42, 122.66, 121.91, 116.41, 23.90; HRMS (ESI) m/z calcd for C_11_H_9_NO_2_ [M + H]^+^ 188.0706, found 188.0705.

*Chemical synthesis of EU-2.* A mixture of DL-tryptophan methyl ester (1.309 g, 6 mmol) and EU-1 (1.123 g, 6 mmol) in methylbenzene (50 mL) was heated to 120^o^C for 2 h. Then the methylbenzene was removed and replaced with 50 mL of dichloromethane. After cooling to 0 ^o^C, 0.5 mL of trifluoroacetic acid was slowly added in batches. The solution was stirred at room temperature until EU-1 was consumed completely. Then the mixture was poured into 7% ammonia water (30 mL) and diluted with 30 mL of ethyl acetate. After extraction and drying, racemate EU-2 (pale yellow solid, 5.502 g, yield 98%) was obtained by silica gel chromatography. HRMS (ESI) m/z calcd for C_23_H_21_N_3_O_3_ [M + H]^+^ 388.1655, found 388.1653.

*Chemical synthesis of EU-3.* To a stirred solution of EU-2 (1.937 g, 5 mmol) in xylene (60 mL) was added 10% Pd/C (200 mg). The mixture was heated at 150 °C under oxygen atmosphere until EU-2 was consumed completely. After filtration and concentration, EU-3 (white solid, 1.418 g, yield 74%) was obtained by silica gel chromatography. m.p. 192.3–194.1 °C; ^1^H NMR (500 MHz, DMSO-*d*_*6*_) *δ* 11.96 (s, 1H), 8.92 (s, 1H), 8.69 (d, *J* = 10 Hz, 1H), 8.62 (s, 1H), 8.50*–*8.46 (m, 2 H), 7.76 (d, *J* = 10 Hz, 1H), 7.66 (t, *J* = 7.5 Hz, 1H), 7.48*–*7.36 (m, 3 H), 3.99 (s, 3 H), 2.91 (s, 3 H); ^13^C NMR (125 MHz, DMSO-*d*_*6*_) *δ* 170.48, 166.51, 141.69, 137.67, 136.87, 135.85, 134.98, 129.48, 129.40, 129.25, 127.69, 125.96, 124.48, 123.24, 122.55, 121.93, 121.12, 118.21, 116.59, 116.27, 113.13, 52.66, 24.65. HRMS (ESI) m/z calcd for C_23_H_17_N_3_O_3_ [M + H]^+^ 384.1342, found 384.1345.

*Chemical synthesis of EU-4.* To a stirred solution of EU-3 (1.534 g, 4 mmol) in anhydrous ethanol (30 mL) was added anhydrous calcium chloride (44 mg, 0.4 mmol) powder in batches. The mixture was stirred at room temperature until complete conversion. Then the reaction was quenched with water. The mixture was diluted with water (50 mL) and ethyl acetate (50 mL). After extraction and drying, EU-4 (creamy white solid, 1.091 g, yield 87%) was obtained by silica gel chromatography. ^1^H NMR (500 MHz, DMSO-*d*_*6*_) *δ* 11.67 (s, 1H), 11.18 (s, 1H), 8.63 (d, *J* = 5 Hz, 1H), 8.30 (d, *J* = 5 Hz, 1H), 8.24 (d, *J* = 10 Hz, 1H), 8.00 (s, 1H), 7.68 (d, *J* = 5 Hz, 1H), 7.54*–*7.50 (m, 2 H), 7.25*–*7.20 (m, 2 H), 7.14 (t, *J* = 7.5 Hz, 1H), 5.34 (t, *J* = 7.5 Hz, 1H), 4.84 (d, *J* = 5 Hz, 2 H); ^13^C NMR (125 MHz, DMSO-*d*_*6*_) *δ* 150.52, 141.63, 139.57, 137.04, 131.44, 129.55, 128.03, 126.77, 126.40, 123.07, 122.49, 121.87, 121.73, 120.25, 119.76, 113.70, 112.87, 111.96, 108.19, 65.44. The NMR spectra could be found in Fig. S1. HRMS (ESI) m/z calcd for C_20_H_16_N_3_O [M + H]^+^ 314.1288, found 314.1287.

*Chemical synthesis of EU-5.* To a stirred solution of EU-4 (1.096 g, 3.5 mmol) in 30 mL of anhydrous dichloromethane was added Dess-Martin periodinane (2.969 g, 7 mmol) in batches under ice bath conditions. The solution was stirred at room temperature until EU-4 was consumed completely. Then the saturated sodium bicarbonate solution and the excess sodium thiosulfate solution are added successively. After extraction and drying, EU-5 (pale yellow solid, 0.926 g, yield 85%) was obtained by silica gel chromatography with dichloromethane and methanol (v/v = 19/1) as eluent. ^1^H NMR (500 MHz, DMSO-*d*_*6*_) *δ* 10.25 (s, 1H), 8.77 (d, *J* = 10 Hz, 1H), 8.68 (s, 1H), 8.43*–*8.41 (m, 2 H), 7.99 (d, *J* = 10 Hz, 1H), 7.62 (t, *J* = 7.5 Hz, 1H), 7.56 (d, *J* = 5 Hz, 1H), 7.36 (t, *J* = 7.5 Hz, 1H), 7.26 (t, *J* = 5 Hz, 1H), 7.20 (t, *J* = 5 Hz, 1H); ^13^C NMR (125 MHz, DMSO-*d*_*6*_) *δ* 193.11, 143.01, 141.17, 140.68, 136.50, 134.20, 128.33, 128.19, 126.81, 126.68, 126.07, 122.36, 122.23, 121.68, 120.45, 120.09, 112.86, 112.16, 111.89, 111.58. The NMR spectra could be found in Fig. S2. HRMS (ESI) m/z calcd for C_20_H_14_N_3_O [M + H]^+^ 312.1131, found 312.1133.

### Cell lines and cell culture

Dulbecco’s Modified of Eagle’s Medium (DMEM, Gibco) and Roswell Park Memorial Institute medium (RPMI 1640, Gibco) containing 10% fetal bovine serum were used to culture cells at 37 ^o^C in a humidified atmosphere of 5% CO_2_ [4].

### Cell viability assay

As reported previously, MTT (Solarbio) method was used to screen cytotoxicity [4]. Briefly, 4 × 10^3^ cells were seeded in 96-well plates per well. Then compound EU-5 was added the next day and treated for 48 h. And the final concentration of compound EU-5 was set as 10 *µ*M. In addition, CCK-8 (Biosharp) assay was carried out to assess the IC_50_ value that EU-5 against A375 cells [22]. Cisplatin and DMSO (0.01%) were used as the positive and negative controls, respectively. Finally, the dose-dependent curve was drawn and the IC_50_ value was calculated.

### Hemolytic toxicity assay

As reported previously [12], fresh rabbits red blood cells (RBCs) were diluted into 2% suspension, which was treated by indicated concentrations of EU-5 (12.5‒800 *µ*M). Triton X-100 (0.1%, Solarbio) and saline were used as positive and negative controls, respectively. The OD_540nm_ value of the supernatant was recorded after the suspension was incubated at 37 ^o^C for 1 h.

### Physicochemical properties calculation

As reported previously [14], the physicochemical properties of EU-5 were calculated in the webpage SwissADME (http://www.swissadme.ch/), which were displayed in Table S1.

### Colony formation assay

As reported previously [34], the A375 cells were seeded in 6-well plates with the mount of 1 × 10^3^ cells/well. After incubation at 37 ^o^C for 24 h, the medium was replaced with indicated concentrations of EU-5 (0, 1.1, 2.2 and 4.4 *µ*M). Specially, the medium was replaced every three day. After a week of continuous cultivation, colonies were fixed with 4% paraformaldehyde solution and stained for 30 min with crystal violet solution (Beyotime). Finally, the colonies were counted by ImageJ and analyzed by Student’s t-test.

### EdU assay

As reported previously [17], the A375 cells (3 × 10^5^ cells/well) were seeded in 6-well plates and cultured for 24 h. Then the DMEM medium was replaced with medium containing compound EU-5 (0 and 4.4 *µ*M). After incubation for 48 h, BeyoClick™ EdU Cell Proliferation Kit with Alexa Fluor 594 (Beyotime) was used to evaluate the proliferation inhibition activity. The 10 *µ*M of EdU was added to A375 cells and cultured at 37 ^o^C for 2 h. Then, cells were fixed and incubated for 30 min with the addition of Click reaction buffer. Finally, Hoechst was added for all cell nucleic acid staining. Images were obtained by using the Olympus IX73 inverted fluorescence microscope. Cell proliferation rates were calculated by using the ImageJ software.

### Cell cycle arrest assay

As reported previously [16], the A375 cells (3 × 10^5^ cells/well) were seeded in 6-well plates and cultured for 24 h, then treated by indicated concentrations of EU-5 (0, 1.1 and 2.2 *µ*M) for 48 h. After collection and washing, the A375 cells were fixed by 75% of pre-cooled ethanol at 4 ^o^C for 18 h. Subsequently, the cells were treated by PI staining solution and RNase A (Cell Cycle and Apoptosis Analysis Kit, Meilun), which were cultured at 37 ^o^C for 30 min. Finally, the cell cycle distribution was measured by flow cytometry (BD C6plus). In order to present the experimental results more clearly, the selective analysis of cell populations through the Gate function of ModFit LT 5.0 was carried out.

### Wound healing assay

As reported previously [18], the A375 cells (1 × 10^6^ cells/well) were seeded in 6-well plates and cultured for 24 h. Wounds were created and the suspended cells are washed away. Then the serum-free DMEM medium containing indicated concentrations of EU-5 (0, 2.2 and 4.4 *µ*M) was added and images were obtained using a Nikon Eclipse Ti-s inverted fluorescence microscope, the migration activity was recorded and analyzed by ImageJ software at the specified time node (0, 24 and 48 h).

### Transwell assay

As reported previously [35], the A375 cells (1 × 10^4^ cells/well) were seeded in Transwell insets (8-*µ*m membrane, Corning) which were placed in 24-well plates and the serum-free DMEM medium containing 0 or 4.4 *µ*M of EU-5 was added. The DMEM medium containing 20% FBS was added in the bottom. After incubation for 24 h, the non-migrated cells on top of the Transwell membrane were removed with a cotton swab. The migrating cells were fixed with paraformaldehyde (4%) and stained with DAPI. Then the migrated cells on the bottom of the Transwell membrane were taken photos by using an inverted fluorescence microscope (Nikon Eclipse Ti-s) and counted from five random fields. The data analyzed by using GraphPad Prism 8.0.2 software.

### Calcein-AM/PI staining assay

As reported previously [19], the A375 cells (5 × 10^4^ cells/well) were seeded in 24-well plates, which were cultured for 24 h and the DMEM medium was replaced with medium containing EU-5 (0 and 4.4 *µ*M). After incubation for 48 h, the cells were stained by using the Calcein-AM/PI Kit (Beyotime). Finally, the cells were taken photos by using an inverted fluorescence microscope (Olympus IX73).

### Cell apoptosis assay

As reported previously [21], the A375 cells (5 × 10^5^ cells/well) were seeded in 6-well plates and incubated for 24 h. Then the DMEM medium containing indicated concentrations of EU-5 (0, 2.2 and 4.4 *µ*M) was added. After incubation for 48 h, the cells were harvested and washed. Then the Annexin V-FITC/PI Apoptosis Kit (Meilumbio) was used to detect cell apoptosis by using flow cytometry.

### MMP assay

As reported previously [22, 23], the A375 cells (5 × 10^5^ cells/well) were seeded in 6-well plates and incubated for 24 h. Then the cells were treated by indicated concentrations of EU-5 (0, 2.2 and 4.4 *µ*M) for 48 h. CCCP (10 *µ*M) was used as positive control. Then the MMP Assay Kit with JC-1 (Beyotime) was used to detect the distribution and change of MMP by using laser confocal microscope (Leica TCS SP8) and flow cytometry, respectively.

### ROS assay

As reported previously [21, 22], the A375 cells (3 × 10^5^ cells/well) were seeded in 6-well plates and cultured for 24 h, which were further treated by EU-5 (0, 2.2 and 4.4 *µ*M) for 48 h. Then the 2′,7′-dichlorofluorescein diacetate (DCFH-DA) solution (10 *µ*M, Beyotime) was added into the treated cells, which were incubated at 37 ^o^C for 20 min. After washing and digestion, the cells were collected and detected by flow cytometer. The data was analyzed by FlowJo VX software.

### Transcriptomics assay

As reported previously [25, 36], 6 mL of A375 cells (5 × 10^4^cells/mL) were seeded in culture dish and cultured for 24 h. Then the cells were treated by 4.4 *µ*M of EU-5 or corresponding DMSO, and three biological replicates were performed. After washing and collection, total RNA was extracted and its purity and integrity was further evaluated by using Agilent 2100 bioanalyzer. Then the mRNA was captured by oligo-dT beads and broken randomly in NEB fragmentation buffer. Subsequently, the library was constructed by the NEB normal library building method. Preliminary quantification was carried out by Qubit 2.0 fluorometer and the effective concentration of the library was accurately quantified by qRT‑PCR, which was greater than 2 nM. Finally, the RNA sequencing was performed by using Illumina system at Wekemo Tech Group Co., Ltd., Shenzhen, China. Moreover, the data was analyzed by using the online platform of Wekemo Bioincloud (https://www.bioincloud.tech/). The Padj < 0.05 and |log_2_FC| ≥ 1 were used to confirm the DEGs. The results of GO and KEGG analysis were shown in Table S3 and S4.

### qRT‑PCR assay

As reported previously [26], primers were designed by using Primer 5.0 software as shown in Table S4 and prepared by Sangon Biotech Co., Ltd. (Shanghai, China). Total RBA was extracted by using Trizol (Solarbio) and reverse transcription was performed by using ReverTra Ace® qPCR RT Kit (FSQ-101, TOYOBO). Then qRT‑PCR was carried out by 2× M5 HiPer SYBR Premix EsTag Kit (Mei5bio) to measure the relative expression of target genes relative to GAPDH (By Thermo Fisher QuantStudio™ 5).

### UV absorption and fluorescence spectroscopic assay

As reported previously [32], the UV and fluorescence emission spectra of compound EU-5 (5 × 10^− 5^ M) were titrated by indicated concentrations of ctDNA (0 − 3 × 10^− 5^ M). The UV spectra were recorded between 300 and 500 nm, and the fluorescence emission spectra were recorded between 400 and 700 nm.

### Molecular docking assay

As reported previously [33], the structure of DNA (PDB ID: 1Z3F) were obtained from RCSB protein data bank. The structure of EU-5 was drawn and processed in the Sybyl-X 2.0 package. Docking study was carried out by using the Surflex-Dock program.

### Statistical analysis

Three biological replicates were performed unless stated. Data were expressed as mean ± standard deviation (SD). The significance of difference was analyzed by using GraphPad Prism 8.0 software. The data normality was analyzed by Shapiro-Wilk test and the significant differences among groups were analyzed using the Student’s t-test and one- or two-way ANOVA.

## Conclusion

**Fig. 7:**
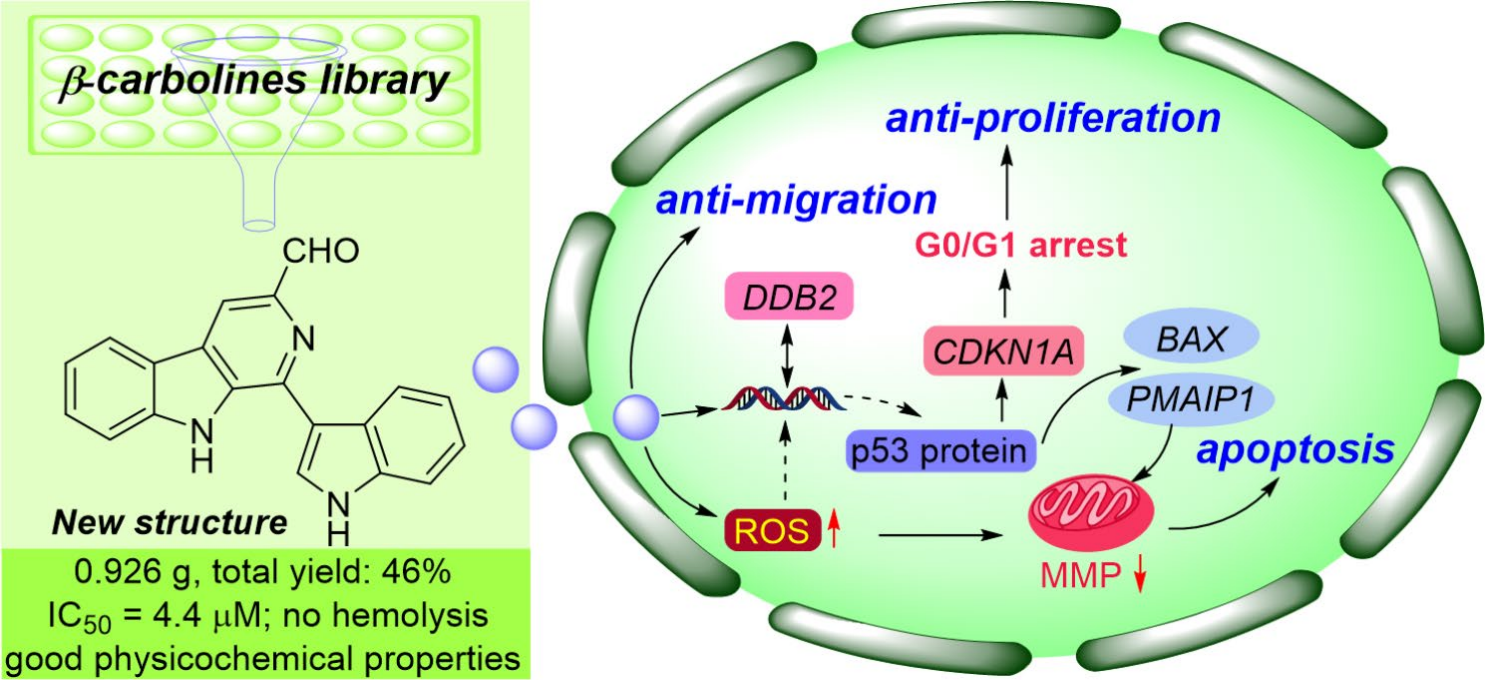
The flow diagram of this work: synthesis, anti-melanoma activity and mechanism of action of 3-formyl-eudistomin U

In this work (Fig. 7), EU-5 was synthesized and its antitumor activity against ten kinds of human cancer cells was evaluated, which exhibited potent anti-melanoma effect with IC_50_ of 4.4 *µ*M. Moreover, no hemolytic toxicity was found and good physicochemical properties *in silico* were presented, which indicated the potential of EU-5 as a lead compound. Further mechanism study revealed that EU-5 could suppress the proliferation of A375 cells by arresting cell cycle at G0/G1 phase and inhibit migration in a dose-dependent manner. The increase of ROS level might directly decrease the mitochondrial transmembrane potential and thus trigger the cell apoptosis. In addition, EU-5 could damage DNA by directly binding or overexpression of intracellular ROS, which further activated the gene expression of *BAX* and *PMAIP1* in p53 signaling pathway and finally triggered mitochondria-mediated cell apoptosis. Overall, EU-5 exerted anti-melanoma activity via a multi-target action mode centered on the p53 signaling pathway. Future structural optimization design targeting DNA damage based on this backbone may discover more drug candidates against malignant melanoma.

### Electronic supplementary material

Below is the link to the electronic supplementary material.

**Supplementary Material 1**: **Fig. S1**: The ^1^H-NMR and ^13^C-NMR spectra of compound EU-4. **Fig. S2**: The ^1^H-NMR and ^13^C-NMR spectra of compound EU-5. **Fig. S3**: The in vitro antitumor activity of cisplatin against A375 cells. **Fig. S4**: The relative viability of HTR-8 cells treated by indicated concentrations of EU-5 and cisplatin. **Fig. S5**: The forming colonies were counted by Image J software and quantified by histogram. **Fig. S6**: A375 cells in EdU treated with 4.4 µM of EU-5 for 48 h. ***P < 0.001 vs control group. **Fig. S7**: The volcano plots of the DEGs. The 175 DEGs were up-regulated and 198 DEGs were down-regulated. **Table S1**: Physicochemical properties of 3-formyl-eudistomin U in silico. **Table S2**: The enriched GO terms of DEGs. **Table S3**: The KEGG enrichment analysis of DEGs. Table S4 The primer sequences used in this study.

## Data Availability

Data will be available on request by the corresponding author.
