# Supplementary material for: Synthesis of 3-formyl-eudistomin U with anti-proliferation, anti-migration and apoptosis-promoting activities on melanoma cells
Source: BMC Chem. 2023 Dec 20;17(1):184. doi: 10.1186/s13065-023-01102-1 (PMC10734049; doi:10.1186/s13065-023-01102-1)
Supplement: Supplementary file 1 — Supplementary Material 1: Fig. S1: The 1H-NMR and 13C-NMR spectra of compound EU-4. Fig. S2: The 1H-NMR and 13C-NMR spectra of compound EU-5. Fig. S3: The in vitro antitumor activity of cisplatin against A375 cells. Fig. S4: The relative viability of HTR-8 cells treated by indicated concentrations of EU-5 and cisplatin. Fig. S5: The forming colonies were counted by Image J software and quantified by histogram. Fig. S6: A375 cells in EdU treated with 4.4 µM of EU-5 for 48 h. ***P < 0.001 vs control group. Fig. S7: The volcano plots of the DEGs. The 175 DEGs were up-regulated and 198 DEGs were down-regulated. Table S1: Physicochemical properties of 3-formyl-eudistomin U in silico. Table S2: The enriched GO terms of DEGs. Table S3: The KEGG enrichment analysis of DEGs. Table S4 The primer sequences used in this study. [file 13065_2023_1102_MOESM1_ESM.pdf]

Supporting Information

Synthesis of 3-formyl-eudistomin U with anti-proliferation, anti-migration and apoptosis-promoting activities on melanoma cells

Jixiang Gao[1,2,†], Jinyi Liu[1,†], Tao Yu[1,†], Chenggong Xu[1], Hao Sun[1], Chunbo Lu[1], Wenjia Dan[1,*], and Jiangkun Dai[1,*]

[1]*School of Life Science and Technology, Weifang Medical University, Weifang 261053, Shandong Province, China.*

[2]*Central Hospital Affiliated to Shandong First Medical University, Jinan 250100, Shandong Province, China.*

[†]These authors contribute equally to this work.

*Corresponding author

Dr. Jiangkun Dai,

E-mail: daijkun@hotmail.com/daijkun@wfmc.edu.cn

Dr. Wenjia Dan,

E-mail: dwj586@163.com

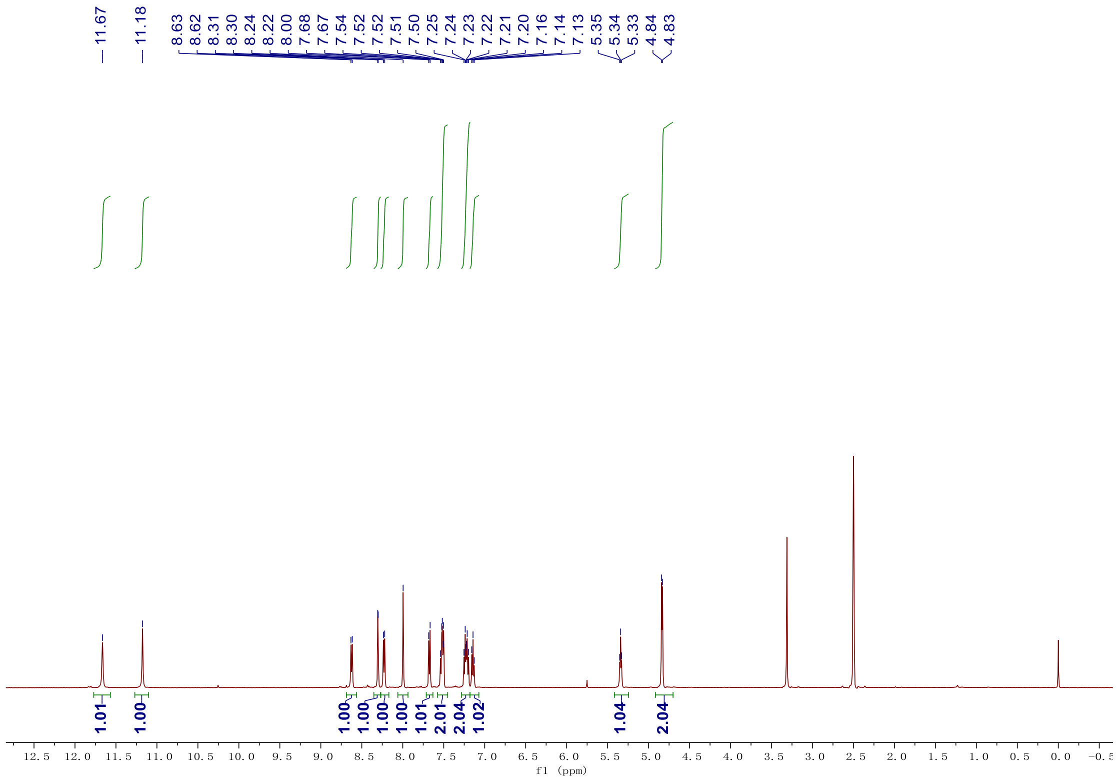

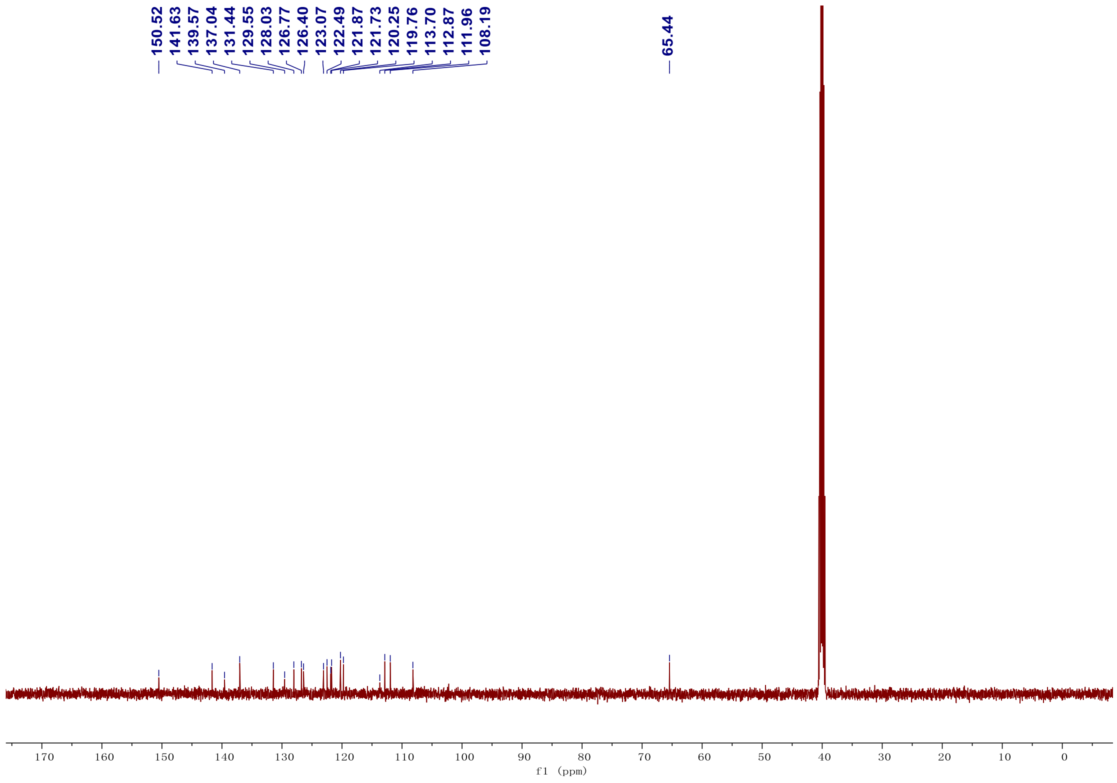

Fig. S1 The ^{1}H-NMR and ^{13}C-NMR spectra of compound EU-4.

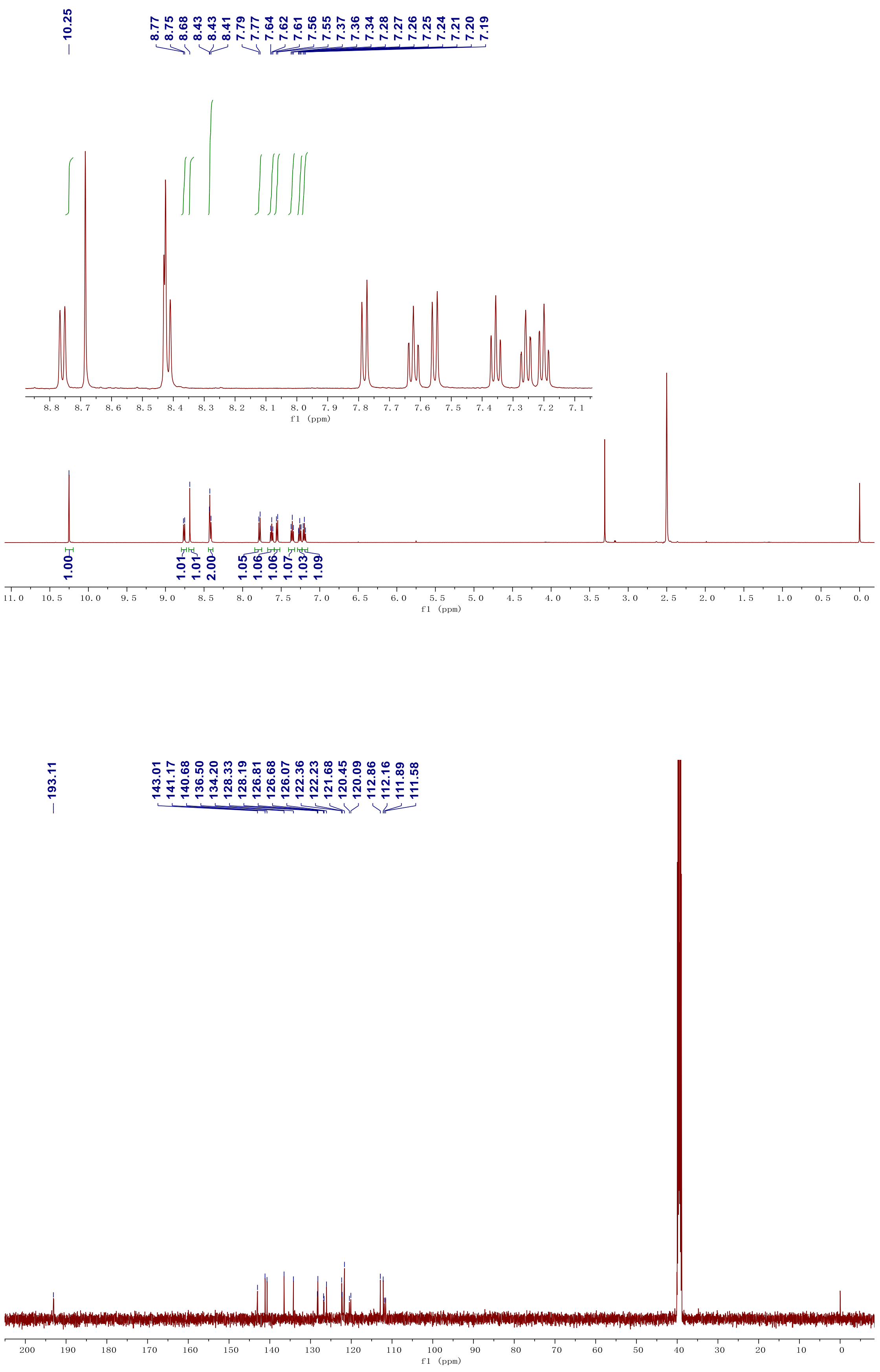

Fig. S2 The ^{1}H-NMR and ^{13}C-NMR spectra of compound EU-5.

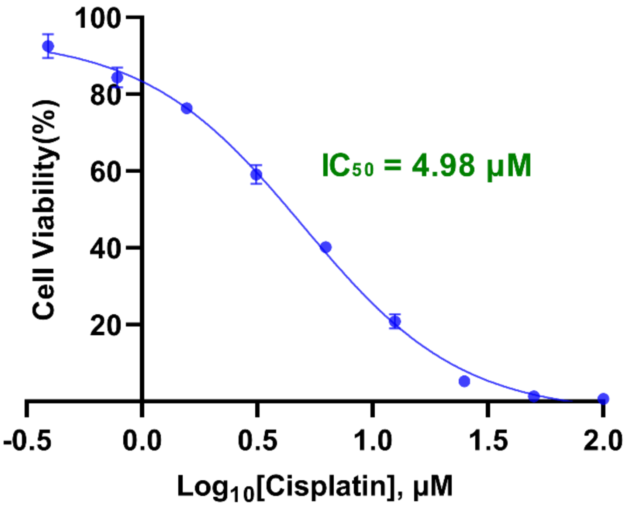

Fig. S3 The *in vitro* antitumor activity of cisplatin against A375 cells.

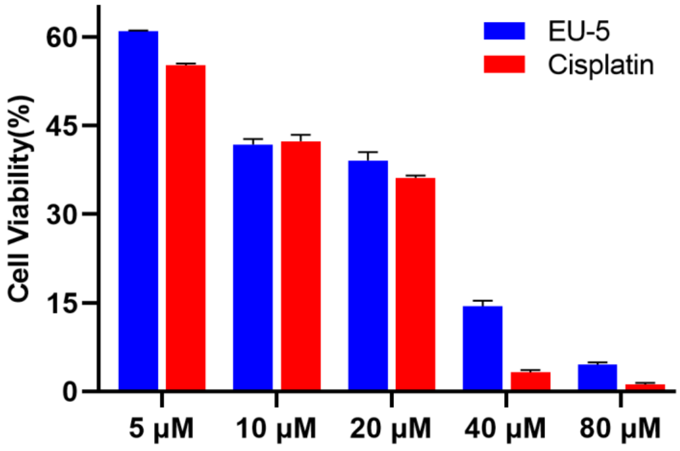

Fig. S4 The relative viability of HTR-8 cells treated by indicated concentrations of EU-5 and cisplatin.

Table S1 Physicochemical properties of 3-formyl-eudistomin U *in silico*.

MW	Log$P_{o/w}$	HBD	HBA	RBN	GI	P-gp	CYP2D6
311.34	3.5	2	2	2	High	Inhibitor	Non-inhibitor

MW: molecular weight; Log$P_{o/w}$: octanol–water partition coefficient; HBD: number of hydrogen bond donors; HBA: number of hydrogen bond acceptors; RBN: number of rotatable bonds; GI: gastrointestinal absorption; P-gp (permeability glycoprotein) suggested the most important member among ATP-binding cassette transporters or ABC-transporters; CYP2D6 (cytochrome P450 2D6) predicted CYP2D6 enzyme inhibition using 2D chemical structure.

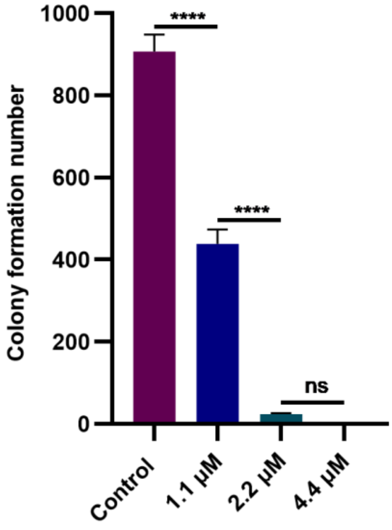

Fig. S5 The forming colonies were counted by Image J software and quantified by histogram.

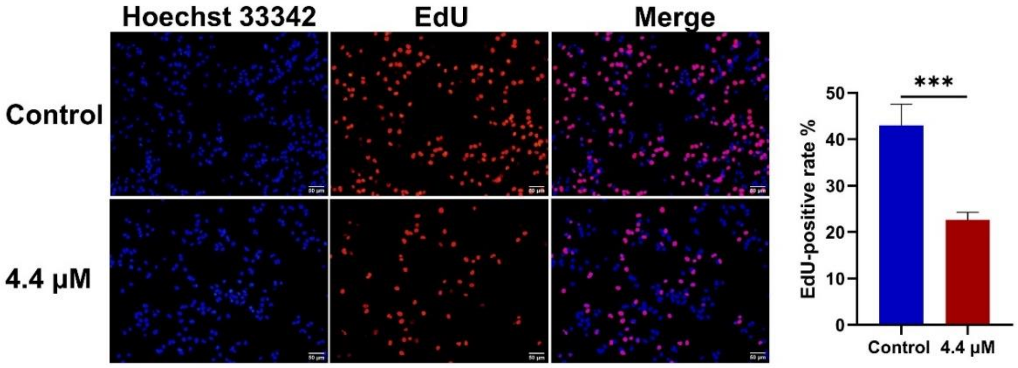

Fig. S6 A375 cells in EdU treated with 4.4 μM of EU-5 for 48 h. ***$P < 0.001$ *vs* control group.

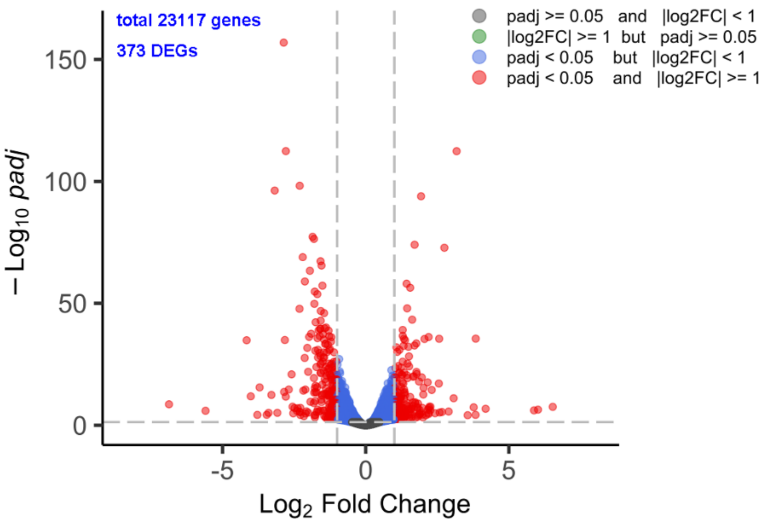

Fig. S7 The volcano plots of the DEGs. The 175 DEGs were up-regulated and 198 DEGs were down-regulated.

Table S2 The enriched GO terms of DEGs.

**Term_type**	**GO_accession**	**GO_term**	**qvalue**
Biological process	GO:0006986	response to unfolded protein	2.94×10^{-9}
Biological process	GO:0034620	cellular response to unfolded protein	2.24×10^{-8}
Biological process	GO:0034976	response to endoplasmic reticulum stress	4.36×10^{-6}
Biological process	GO:0009636	response to toxic substance	0.0001
Biological process	GO:0097193	intrinsic apoptotic signaling pathway	0.0002
Biological process	GO:0006520	cellular amino acid metabolic process	0.0003
Biological process	GO:0030330	DNA damage response, signal transduction by p53 class mediator	0.0005
Biological process	GO:1902475	L-alpha-amino acid transmembrane transport	0.0009
Biological process	GO:0007050	cell cycle arrest	0.0014
Biological process	GO:0033273	response to vitamin	0.0019
Biological process	GO:2000045	regulation of G1/S transition of mitotic cell cycle	0.0020
Biological process	GO:0042026	protein refolding	0.0021
Biological process	GO:0001822	kidney development	0.0021
Biological process	GO:0008585	female gonad development	0.0029
Biological process	GO:1905897	regulation of response to endoplasmic reticulum stress	0.0039
Biological process	GO:0051085	chaperone cofactor-dependent protein refolding	0.0041
Biological process	GO:0050673	epithelial cell proliferation	0.0043
Biological process	GO:0044774	mitotic DNA integrity checkpoint	0.0044
Biological process	GO:1902806	regulation of cell cycle G1/S phase transition	0.0044
Biological process	GO:0033598	mammary gland epithelial cell proliferation	0.0058
Biological process	GO:0000082	G1/S transition of mitotic cell cycle	0.0058
Biological process	GO:0007568	aging	0.0067
Biological process	GO:0061077	chaperone-mediated protein folding	0.0072
Biological process	GO:0010165	response to X-ray	0.0072

Biological process	GO:0035767	endothelial cell chemotaxis	0.0072
Biological process	GO:0044783	G1 DNA damage checkpoint	0.0073
Biological process	GO:0098657	import into cell	0.0076
Biological process	GO:0031110	regulation of microtubule polymerization or depolymerization	0.0076
Biological process	GO:0043648	dicarboxylic acid metabolic process	0.0085
Biological process	GO:0044843	cell cycle G1/S phase transition	0.0106
Biological process	GO:0048732	gland development	0.0111
Biological process	GO:0031334	positive regulation of protein-containing complex assembly	0.0117
Biological process	GO:0006418	tRNA aminoacylation for protein translation	0.0120
Biological process	GO:0140467	integrated stress response signaling	0.0121
Biological process	GO:2001233	regulation of apoptotic signaling pathway	0.0123
Biological process	GO:0043536	positive regulation of blood vessel endothelial cell migration	0.0124
Biological process	GO:1904018	positive regulation of vasculature development	0.0173
Biological process	GO:0072593	reactive oxygen species metabolic process	0.0178
Biological process	GO:0009314	response to radiation	0.0188
Biological process	GO:0045667	regulation of osteoblast differentiation	0.0208
Biological process	GO:0042594	response to starvation	0.0222
Biological process	GO:0046890	regulation of lipid biosynthetic process	0.0222
Biological process	GO:0001649	osteoblast differentiation	0.0229
Biological process	GO:0051091	positive regulation of DNA-binding transcription factor activity	0.0229
Biological process	GO:1903825	organic acid transmembrane transport	0.0229
Biological process	GO:0032075	positive regulation of nuclease activity	0.0244
Biological process	GO:0045444	fat cell differentiation	0.0246
Biological process	GO:0034332	adherens junction organization	0.0246
Biological process	GO:0060688	regulation of morphogenesis of a branching structure	0.0246
Biological process	GO:0045598	regulation of fat cell differentiation	0.0267

Biological process	GO:0003206	cardiac chamber morphogenesis	0.0279
Biological process	GO:0098739	import across plasma membrane	0.0286
Biological process	GO:0043542	endothelial cell migration	0.0291
Biological process	GO:0045446	endothelial cell differentiation	0.0299
Biological process	GO:0030195	negative regulation of blood coagulation	0.0299
Biological process	GO:0038065	collagen-activated signaling pathway	0.0299
Biological process	GO:0090200	positive regulation of release of cytochrome c from mitochondria	0.0299
Biological process	GO:0031109	microtubule polymerization or depolymerization	0.0303
Biological process	GO:1901617	organic hydroxy compound biosynthetic process	0.0312
Biological process	GO:1900047	negative regulation of hemostasis	0.0312
Biological process	GO:0019934	cGMP-mediated signaling	0.0321
Biological process	GO:0060445	branching involved in salivary gland morphogenesis	0.0321
Biological process	GO:0090130	tissue migration	0.0338
Biological process	GO:1903035	negative regulation of response to wounding	0.0343
Biological process	GO:0010763	positive regulation of fibroblast migration	0.0343
Biological process	GO:0055057	neuroblast division	0.0343
Biological process	GO:0002064	epithelial cell development	0.0343
Biological process	GO:0051098	regulation of binding	0.0343
Biological process	GO:0050819	negative regulation of coagulation	0.0371
Biological process	GO:0000302	response to reactive oxygen species	0.0378
Biological process	GO:0051775	response to redox state	0.0378
Biological process	GO:0060576	intestinal epithelial cell development	0.0378
Biological process	GO:0010721	negative regulation of cell development	0.0385
Biological process	GO:0042445	hormone metabolic process	0.0386
Biological process	GO:0031960	response to corticosteroid	0.0387
Biological process	GO:0006766	vitamin metabolic process	0.0387

Biological process	GO:0050678	regulation of epithelial cell proliferation	0.0410
Biological process	GO:0002262	myeloid cell homeostasis	0.0410
Biological process	GO:0034644	cellular response to UV	0.0410
Biological process	GO:0070365	hepatocyte differentiation	0.0412
Cellular component	GO:0045178	basal part of cell	0.0277
Cellular component	GO:0060076	excitatory synapse	0.0277
Cellular component	GO:0009925	basal plasma membrane	0.0300
Cellular component	GO:0062023	collagen-containing extracellular matrix	0.0451
Cellular component	GO:0060198	clathrin-sculpted vesicle	0.0451
Molecular function	GO:0044183	protein folding chaperone	3.31×10^{-6}
Molecular function	GO:0015179	L-amino acid transmembrane transporter activity	0.0010
Molecular function	GO:0051787	misfolded protein binding	0.0011
Molecular function	GO:0008201	heparin binding	0.0011
Molecular function	GO:1901681	sulfur compound binding	0.0011
Molecular function	GO:0008483	transaminase activity	0.0014
Molecular function	GO:0005539	glycosaminoglycan binding	0.0025
Molecular function	GO:0031072	heat shock protein binding	0.0073
Molecular function	GO:0004812	aminoacyl-tRNA ligase activity	0.0073
Molecular function	GO:0016875	ligase activity, forming carbon-oxygen bonds	0.0073
Molecular function	GO:0050840	extracellular matrix binding	0.0100
Molecular function	GO:0001618	virus receptor activity	0.0172
Molecular function	GO:0140272	exogenous protein binding	0.0172
Molecular function	GO:0019210	kinase inhibitor activity	0.0308
Molecular function	GO:0055131	C3HC4-type RING finger domain binding	0.0344
Molecular function	GO:0048531	beta-1,3-galactosyltransferase activity	0.0400
Molecular function	GO:0051082	unfolded protein binding	0.0400

Table S3 The KEGG enrichment analysis of DEGs.

**Term_ID**	**Gene_Ratio**	**Pathway**	**qvalue**
hsa01230	12/173	Biosynthesis of amino acids	5.04×10^{-8}
hsa04115	9/173	p53 signaling pathway	0.0025
hsa00260	6/173	Glycine, serine and threonine metabolism	0.0137
hsa01210	4/173	2-Oxocarboxylic acid metabolism	0.0339
hsa00250	5/173	Alanine, aspartate and glutamate metabolism	0.0406
hsa00220	4/173	Arginine biosynthesis	0.0406

Table S4 The primer sequences used in this study.

Gene	Forward primer (5′ → 3′)	Reverse primer (5′ → 3′)
CDKN1A	TGTCCGTCAGAACCCATGC	AAAGTCGAAGTTCCATCGCTC
BAX	CCCGAGAGGTCTTTTTCCGAG	CCAGCCCATGATGGTTCTGAT
PIDD1	TCAGAGGATTCGGACGCAG	GTGAGTGCTCAGACGCAAGAA
SESN1	TGCTTTGGGCCGTTTGGATAA	TGTAGTGACGATAATGTAGGGGT
THBS1	AGACTCCGCATCGCAAAGG	TCACCACGTTGTTGTCAAGGG
DDB2	ACCTCCGAGATTGTATTACGCC	TCACATCTTCTGCTAGGACCG
SESN2	AAGGACTACCTGCGGTTCG	CGCCCAGAGGACATCAGTG
GAPDH	GGAGCGAGATCCCTCCAAAAT	GGCTGTTGTCATACTTCTCATGG
